# Radioguided localisation of non-palpable lesions of the breast in Costa Rica: review of results of our first 800 patients in private practice

**DOI:** 10.3332/ecancer.2017.745

**Published:** 2017-06-08

**Authors:** Marisel Aguilar, Sabrina Alfaro, Ricardo Aguilar

**Affiliations:** 1 Division of Breast Surgery, Clinica Biblica Hospital, San José, Costa Rica; 2 Division of Surgery, Hospital of Greece, Alajuela, Costa Rica; 3 Division of Intraoperative Radiotherapy, Clinica Biblica Hospital, Calle central y primera, Avenidas 14 16, Apartado 1307-1000 San José, Costa Rica

**Keywords:** breast neoplasm, breast cancer, localisation, immunoscintigraphy detection

## Abstract

**Background:**

Surgical treatment of non-palpable breast lesions is controversial. At the European Institute of Oncology in Milan, Italy, Prof Umberto Veronesi introduced a new technique called the radioguided occult lesion localisation (ROLL) in 1996 to replace conventional methods and their disadvantages (Zurrida S, Galimberti V, and Monti S *et al* (1998) **Radioguided localization of occult breast lesions** *Breast* **7** 11–13 https://doi.org/10.1016/S0960-9776(98)90044-3). Given the success experienced in that institution, the method became the technique of choice for the early diagnosis of breast cancer. In this paper, we will examine the technical aspects of ROLL and the results from a large series of patients treated in our private practice in Costa Rica.

**Methods:**

We analysed the first 816 patients with different non-palpable breast lesions detected by ultrasound or mammography within our private practice in Costa Rica. In 774 patients, technetium 99m labelled with human serum albumin (7–10 MBq) in 0.2 ml of saline solution was injected into the lesion under mammographic or ultrasound guidance. The excisional biopsy was done by means of a gamma-probe and complete excision of the lesion was verified by X-ray on the specimen in lesions that were visible by mammography and ultrasound 4 months after surgery. In the remaining 42 patients, the localisation of the lesion was carried out by wire.

**Results:**

The tracer was correctly positioned in the first attempt in 772/816 (94.6%) of cases and in the second attempt in two other cases. In 42/816 (5.1%) cases, the localisation of the lesion had to be performed with the traditional method. X-rays showed that the lesion was entirely removed in 770/772 (99.74%) of cases.

**Conclusion:**

The ROLL is a simple and excellent option for the removal of hidden breast lesions in clinical practice. It offers the advantage of making resections safer and with tumour-free margins, in addition to reducing the number of reinterventions. Since it makes it possible to specify to the pathologist the exact site where the lesion is located, we can guarantee a better diagnosis. The rate of success with the use of this technique corresponds to the available scientific data, so we conclude that it is a procedure that we can routinely perform in private practice in Costa Rica.

## Introduction

The increased use of early breast cancer screening tests, such as mammography and ultrasound (US), has increased the number of cases of non-palpable breast lesions, which may eventually be invasive or *in situ* carcinomas. Recent data indicate that 15–25% of breast cancers are intraductal or *in situ*, and in most of these, the lesions are clinically occult [1–9]. A fundamental step in the surgery of these lesions is pre-operative localisation. Safe localisation (site of marking less than 1 m from the lesion) increases the likelihood of radical tumour excision with minimal cosmetic damage and tumour-free margins.

The most commonly used methods in pre-operative marking of non-palpable breast lesions are the injection of carbon particles or hooked wire insertion, but these procedures have several disadvantages [6–8].

- The presence of carbon particles can be problematic for the histological evaluation of the tissue.
- The wire is an invasive, traumatic technique that can lead to bleeding, infection and pneumothorax. The fact that it is disruptive in some cases also limits its effectiveness [3, 5, 7].
- The use of the wire has a high incidence of residual disease at the biopsy site and therefore generates more second interventions [2].

In view of this, our team decided to implement the radiotracer technique for locating non-palpable breast lesions. This paper describes in detail the ROLL method and the results obtained in an important series of patients.

## Patients

We performed the ROLL in 774 women with non-palpable breast abnormalities at clinical examination in our private practice from October 2010 to September 2014. The anomalies were identified by mammography or ultrasound for patients with multicentre and multifocal lesions. Pregnant or lactating women were excluded, as well as those who presented microcalcifications. No other type of localisation was used during that period, except in cases where ROLL was unsatisfactory.

## Localisation of the lesion

The localisation was performed on the day before the surgery by the nuclear medicine team. 0.5 μg of human serum albumin macroaggregates were injected at the centre of the lesion. The particle size ranged from 10 to 150 μm (Macrotec, Amershan Nycomed Sorin, Italy) labelled with 7–10 MBq of 99 mTC. These are diluted in 0.2 ml of saline solution, under ultrasonographic guidance or mammography. Marking and quality control were performed according to the manufacturing instructions.

For microcalcifications, opacities or other abnormalities were revealed by mammography and not by US, the mammography equipment used was Senographe DMR, GE Medical Systems. Mammograms were carried out with the Rx tubes oriented first at 15° and then at −15° for reference, and from those third-dimensional images, the coordinates of the lesion were calculated using the computerised system. The craniocaudal view was used for lesions in the upper quadrants. For external or internal lesions, lateral views were required. A 22-G spinal needle, mounted in the stereotactic frame, was inserted into the lesions where the tip of the needle corresponded to the calculated coordinates. The tip of the needle was located in the centre of the lesion and corroborated by a new mammogram. The chuck was removed, and immediately afterwards, the 0.2 ml of radiopaque contrast medium was injected. The needle was removed, and after 5 min, a standard orthogonal mammography was performed to verify the correct localisation of the contrast medium in the lesion. The extent of the contrast medium field and lesion were observed in the last mammogram. If the overlap was not precise or the distance between the opaque medium and the lesion was greater than 2 cm, the localisation was considered to be unsuccessful and was repeated using a different technique (usually the hooked wire) [10, 11].

When the occult lesions were detected by ultrasound alone, the radiotracer was injected under ultrasonographic guidance. The ultrasound examination was done with linear probes at a frequency of 10–13 or 7.5–10 MHz depending on the size of the breast. Subsequently, another probe (7.5–10 MHz) was used for needle biopsy. The needle was placed on the needle biopsy instrument and inserted into the breast manually. The tip of the needle was positioned at the centre of the lesion which was signalled by changes in echogenicity at the site of the lesion. The radiotracer was then injected, followed by 2 ml of saline solution.

For the visible lesions both in ultrasound and in mammography, the tracer was preferably injected by means of ultrasound, since this allows us to find the centre of the lesion more accurately. An ink mark was made on the skin above the lesion as a guide in surgery [10, 11].

## Surgery

Most surgeries were performed under general anaesthesia. The skin incision was made at the level of the areola where the lesion allowed or where the skin was marked, following cosmetic criteria. Radioactivity detected by the gamma probe (GS), (Europrobe, EURORAD, France). The GS was used to check the position of the hot spot required for excision and to decide the necessary resection margins around the hot spot (where the count range falls to a minimum of 1–1.5cps). After removal of the lesion, the surgical site was checked for residual activity greater than control. If it was present, the resection was enlarged. In case of microcalcifications, the specimen was marked with clips in one or more margins and the complete resection and the concentricity of the lesion were verified by means of X-rays. When microcalcifications were considered close to the margins and/or not correctly centred by the surgeon, radicalisations of the margins were performed.

In the case of non-palpable lesions detected by the US, the specimen was marked with ink by the pathologist and cut to verify the presence of the lesion.

## Pathology

The review by freezing was only used in solid non-palpable lesions larger than 1 cm, prepared with hematoxylin/eosin sections. If an invasive carcinoma was diagnosed, resection was expanded to a quadrantectomy. The histological classification was consistent with the Rosen and Oberman modification of the WHO classification.

## Results

The mean age of the 816 consecutive patients was 53 years (range 25–79). Of the 816 occult lesions (4 bilateral), 649 (79.53%) were clusters of microcalcifications found by mammography, and the radiotracer was injected under stereotactic guidance. In 167 cases (20.4%), the lesions were detected by US, and the radiotracer was injected by ultrasonographic guide. In general, the procedure (radiotracer injection) was well tolerated, except in one patient who had a minor allergic reaction, which consisted of a local transient rash, which resolved spontaneously. There were no systemic allergic reactions.

The radiotracer was correctly positioned in all 167 injections performed under the ultrasonographic control, mainly because of the correct position of the first needle. Subsequently, the tracer injection is checked in real time as a change in echogenicity at the site of the lesion.

Of the 649 sites made with stereotactic control, the point between the position of the lesion and that of the radiopaque medium was well located in 627 cases (96.61%), whereas in the remaining 22 cases, which were not sufficiently matched, the patients were sent for another preoperative localisation procedure. Most of the unsuccessful cases presented during the first two years of the study may be due to the surgeons’ learning curve when faced with a new technique.

A total of 794 lesions were referred to scintigraphy. The images showed that the tracer was concentrated in a small, limited area in 619/794 cases (78%). In 20 of the 175 remaining cases, the stereotactic sites were in the central quadrant, the tracer was spread over a large area of the mammary parenchyma, and the localisation was repeated with the wire method. The scan revealed that there was no radioactivity on two lesions (the injection used in stereotaxic surgery not had been correctly performed), and the procedure was successfully repeated in its entirety.

In the 153 cases of minimal contamination, this was present along the path of the needle. The contamination did not interfere with the removal of the lesion, and it was in the process of disappearing by the time of surgery. Skin contamination was found in 5/794 (0.6%) of the cases and did not interfere with the biopsy, and 774/816 lesions were referred to the surgeon as being correctly localised.

In the 100 cases in which the scan was repeated after 5–8 h. There were no differences between the two sets of images in 98 cases. In two patients in whom the tracer was accidentally injected into a galactophore duct, the delayed images showed a different distribution pattern, with a large area of contamination.

The hot spot was located by the GDP in all cases; In 770/774 (99.5%) cases, the subsequent x-rays after excision showed that the lesion contained completely the same with sufficient healthy margins of tissue (greater than 1 cm). In the remaining four cases, the lesion was near or over the margin and a larger excision was necessary. In all these cases, an X-ray of the removed material showed the complete excision of the lesion.

Invasive carcinoma was found in 407/774 (52.6%) of cases, and benign lesions in 367 (47.4%). A total of 405 cancer patients underwent conservative surgery, and two of the patients who were found to have multifocal cancers were mastectomised.

## Discussion

The most commonly used methods for localising non-palpable breast lesions are radiolocalisation, wire insertion, and radiolocalisation with labelled seeds [13, 14]. However, these two latter techniques have their setbacks, including complications such as incomplete removal of the lesion [15].

Previously published results of the ROLL indicated that this method is superior to localisation with a wire, because it provides better centring of the lesion within the specimen and reduces the excision of healthy tissue [15]. However, the main advantage is that it provides the surgeon with a quick and simple localisation for removal of the lesion in the operating room.

The wealth of experience reported by the European Institute of Oncology in Milan largely confirms the results of the comparative studies and amply justifies the acceptance of the ROLL by our country’s surgeons [16]. When the lesion was localised by ultrasonographic guidance, the procedure was simple and fast (5–10 minutes maximum), with an excellent correspondence in all cases between the location of the hot spot and the position of the lesion. The changes in echogenicity caused by the presence of both the needle and the tracer made it possible to verify the correct insertion of the needle tip into the lesion and the correct injection of the tracer.

When stereotaxis was used to guide the tracer site, the needle was not always inserted to the correct depth. The distance between the radiopaque injection site and the lesion was always checked with a standard mammography taken after the injection. When this distance was not greater than 2 cm, the surgeon, with knowledge of the extension and direction of the wrong point, did not have difficulty in completely excising the lesion. In 22 cases, the radiopaque/tracer medium was not injected sufficiently close to the lesion to provide the surgeon with assistance. As we know, this procedure requires specialised personnel for correct execution and these deviations can be explained by the surgeons’ learning curve.

The Gammagram showed problems in another 22 cases:

- In 20 patients, the radiotracer was left inside the ducts or in a lymphatic vessel, so the tracer was diffused, occupying a large part of the breast. In these cases, the localisation was repeated using another method. In 18 of these, the lesion was located in the central quadrant, where the possibility of this problem is greater.
- In the other two cases, the scintigraphy revealed that the breast had not been injected with radioactivity and the injection was repeated successfully.

These findings illustrate how important the scintigraphy is for checking for stereotaxic-guided injection with certainty. However, it should be noted that in the same way in most cases (774/816, 94.8%), including two successful locations on the second attempt.

Skin contamination was rarely observed (0.6%) and was resolved by washing. Minimal contamination along the needle path was relatively common but did not interfere with the removal of the lesion and it disappeared during surgery. Subsequently, our results showed that albumin macroaggregates do not move from the site of injection and these do not diffuse through the breast tissue around the lesion, providing that the radiotracer has not been introduced into a lymphatic vessel or a galactophore duct.

In a previous study, it was shown that ROLL is safe from the point of view of radiation protection for both patients and hospital staff. In addition, it does not require special protective measures by the surgeon or pathologist.

## Conclusions

This study confirms in practice the experience of a large series of previously published studies [15, 16]. For our team, ROLL allows a safe and rapid excision of hidden breast lesions in most cases, reducing the invasiveness of the biopsy, the operative time and the time under anaesthesia.

It is clear that this technique is appropriate in daily private practice in Costa Rica if there is a team of fully trained professionals who have overcome the learning curve.

We have established as a protocol the use of the ROLL together with the sentinel node biopsy in patients in whom the lesions are clinically hidden and suspected to be malignant. In these cases, if the sentinel node is metastatic, we perform axillary dissection immediately, avoiding subsequent surgery for axillary emptying.
